# Consumption of Sugar-Sweetened or Artificially Sweetened Beverages and Semen Quality in Young Men: A Cross-Sectional Study

**DOI:** 10.3390/ijerph19020682

**Published:** 2022-01-07

**Authors:** Maiken Meldgaard, Nis Brix, Anne Gaml-Sørensen, Andreas Ernst, Cecilia Høst Ramlau-Hansen, Sandra Søgaard Tøttenborg, Karin Sørig Hougaard, Jens Peter Ellekilde Bonde, Gunnar Toft

**Affiliations:** 1 Unit for Applied Public Health, Department of Public Health, Research, Aarhus University, 8000 Aarhus, Denmark; mme@ph.au.dk; 2 Research Unit for Epidemiology, Department of Public Health, Aarhus University, 8000 Aarhus, Denmark; nis.brix@ph.au.dk (N.B.); ags@ph.au.dk (A.G.-S.); aernst@ph.au.dk (A.E.);chrh@ph.au.dk (C.H.R.-H.); 3 Department of Clinical Genetics, Aarhus University Hospital, 8200 Aarhus, Denmark; 4 Department of Urology, Aarhus University Hospital, 8200 Aarhus, Denmark; 5 Department of Occupational and Environmental Medicine, Bispebjerg and Frederiksberg Hospital, 2400 Copenhagen, Denmark; sandra.soegaard.toettenborg@regionh.dk (S.S.T.); Jens.Peter.Ellekilde.Bonde@regionh.dk (J.P.E.B.); 6 Denmark and National Research Centre for the Working Environment, Department of Public Health, University of Copenhagen, 1353 Copenhagen, Denmark; KSH@nfa.dk; 7 Department of Public Health, University of Copenhagen, 1353 Copenhagen, Denmark; 8 Steno Diabetes Center Aarhus, Aarhus University Hospital, 8200 Aarhus, Denmark

**Keywords:** soft drink, semen quality, sperm morphology, non-nutritive sweetener, beverage consumption

## Abstract

Background: Existing literature suggests that frequent consumption of sugar-sweetened drinks may be associated with lower semen quality. Studies performed in mice suggest a dose-response relationship between intake of saccharin or aspartame, two artificial sweeteners, and sperm and testis function. Methods: A cross-sectional study based on data from The Fetal Programming of Semen Quality (FEPOS) Cohort, including 1047 young men (mean age = 19 years) was performed. Each male participant completed an online questionnaire on health, health behavior and diet, and provided a semen sample. The associations between consumption of sugar-sweetened or artificially sweetened beverages (moderate ≥ 3 days/week; infrequent < 3 days/week) and semen quality were analyzed using a multivariable, negative, binomial regression model. Results: Sugar-sweetened or artificially sweetened beverage consumption was not strongly associated with either semen volume, sperm concentration, total sperm count or total motility in young men. The proportion of morphologically normal sperm was 11% lower (0.89 (95% CI 0.76, 1.04)) for moderate (≥3 days/week) consumption of artificially sweetened beverages relative to infrequent (<3 days/week). Conclusion: Consumption of sugar-sweetened or artificially sweetened beverages, at the levels present in this study had limited effect on the measured markers of semen quality in young men.

## 1. Introduction

Low semen quality is a major cause of poor male fecundity [1], which is a public health burden linked to psycho-social distress, increased mortality, and the development of diseases [2,3,4,5,6,7,8]. Consumption of sugar-sweetened beverages is frequent and increasing among both children and adults [9,10]. In a few studies, frequent consumption of sugar-sweetened beverages has been associated with lower semen quality [11,12,13]. Especially the consumption of cola has been linked to lower semen characteristics, including semen volume, sperm concentration, total sperm count, and total motility [14,15,16,17].

High consumption of sugar-sweetened beverages is associated with weight gain and obesity [18,19,20,21], and several studies find obesity to be associated with poor semen quality in men [22,23,24,25,26,27,28,29]. However, only one study explored the potential mediatory role of obesity, and found that body mass index (BMI) modified the association between sugar-sweetened beverage consumption and progressive sperm motility [11]. It has been proposed that the underlying mechanisms of obesity are linked to insulin resistance and metabolic syndrome with the associated generation of oxidative stress, which might affect spermatogenesis, but could also interfere with production and regulation of sex hormones, including testosterone and sex hormone-binding globulin levels [18,21,30,31,32,33].

Two studies investigated artificially sweetened beverages in relation to fertility in men and found no association [12,34]. Consumption of artificially sweetened beverages is not associated with weight gain and obesity in itself, but it may affect gut microbiome, taste receptors and a learned behavior toward preference for a sweet taste [35,36,37]. This might increase the risk of obesity and metabolic syndrome, due to higher intake of sugary foods and drinks, and potentially impact male reproduction, including semen quality. In addition, mice studies have found that exposure to high levels of artificial sweeteners, such as saccharin and aspartame, affects several sperm and testicular parameters negatively and, furthermore, interfered with hormones of the hypothalamic-pituitary-gonadal axis [38,39,40].

Overall evidence of associations between consumption and semen quality is scarce in studies on humans. We, therefore, aimed to investigate the associations between consumption of sugar or artificially sweetened beverages and semen quality characteristics, using a large cohort of young Danish men. We hypothesized that consumption of sugar-sweetened or artificially sweetened beverages was associated with lower semen quality characteristics in young men, and that the effect is mediated by higher BMI.

## 2. Materials and Methods

### 2.1. Study Population

This cross-sectional study was based on data from the Fetal Programming of Semen Quality (FEPOS) cohort, a sub-cohort within the Danish National Birth Cohort [41]. The DNBC recruited more than 92,000 pregnant women during their first trimester, in the years between 1996 and 2002. The women provided information on pregnancy, health behavior, and general health in computer-assisted telephone interviews scheduled around gestational week 12.

**Figure 1:**
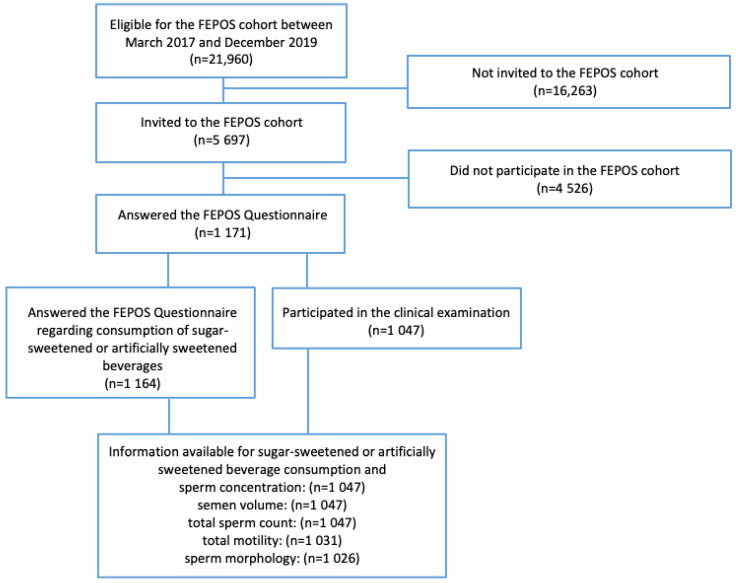
Flowchart of the FEPOS cohort, 2017–2019.

Men eligible for participation in the FEPOS cohort were born between 1998 and 2001 to mothers in the DNBC and lived near Aarhus or Copenhagen at the time they turned 18 years and 9 months of age (*n* = 21,960). Between March 2017–December 2019, a total of 5697 men were selected and invited to participate in the FEPOS cohort [42]. Invited men were encouraged to decline participation if they had a medical history of cryptorchidism, sterilization, or chemotherapy. They filled out a comprehensive online questionnaire to provide in-depth information on diet and health behavior, and information on several anthropometric characteristics, including heigh and weight. In addition, the men delivered a semen sample, a blood sample, and a urine sample. In total, 1047 (19%) participated in both the FEPOS questionnaire and the clinical examination, and had semen characteristics measured, see Figure 1.

### 2.2. Sugar-Sweetened and Artificially Sweetened Beverage Consumption

Information on consumption of sugar-sweetened and artificially sweetened beverages was obtained from the online FEPOS questionnaire. The participants were asked how many days a week (every day; 5–6 days a week; 3–4 days a week; 1–2 days a week; less than one day a week) they consumed sugar-sweetened drinks and artificially sweetened drinks, respectively. Data on sugar-sweetened and artificially sweetened beverage consumption were each dichotomized into moderate (≥3 days a week) and infrequent (<3 days a week) consumption.

### 2.3. Semen Quality Measurement

Participants were instructed to provide a semen sample adjacent to or at the clinical examination. The semen sample was placed on a tilt in an incubator at 37 °C. Participants were encouraged not to ejaculate for 2–4 days before sample collection but were included irrespectively of abstinence time.

Semen analyses were performed manually according to the WHO guidelines [1] and in alignment with the checklist for human semen analysis provided in Björndahl et al. [43]. The two trained biomedical laboratory technicians conducting the semen analyses participated in a follow-up quality control with the Reproductive Medicine Centre in Malmö. The validation was satisfactory and met international standards as well as earlier similar comparisons [42,44].

The following semen characteristics were included in the study: sample volume (mL), concentration (mill./mL) total sperm count (mill. [concentration * volume]), total motility (A + B%), and morphology (% normal sperm). The description of semen characteristics, and analyses, are further described in the FEPOS profile paper [42].

### 2.4. Co-Variates

Prior to analysis, potential confounders were identified using Directed Acyclic Graphs (DAG) [45,46,47] based on existing literature. From the FEPOS questionnaire, we obtained information on the following potential confounders: intake of candy (<3 days a week; ≥3 days a week), intake of cake (<3 days a week; ≥3 days a week), intake of coffee (<3 days a week; ≥3 days a week), intake of energy drink (<3 days a week; ≥3 days a week) and smoking status (yes (yes current, yes occasionally); no (no never, no former)). From the first baseline interview in the DNBC, we obtained information on the following potential confounders: maternal pre-pregnancy BMI (<18.5; 18.5–24.9; ≥25), maternal first trimester smoking status (smoker; non-smoker), and highest parental socioeconomic status (student or unknown; skilled or unskilled worker; low grade professional; high grade professional) based on Danish International Standard Classification of Occupations (DISCO-08), v1:2010 [48].

Other co-variates, including spillage (yes; no), abstinence time (days) and fever within three months prior to sampling (yes; no), were provided by the participants immediately after semen collection, while time to analysis (minutes) was noted by the laboratory technician prior to analysis of the semen sample. The participants’ own BMI was categorized as (<18.5; 18.5–24.9; ≥25 kg/m²) and used in a supplementary analysis. Co-variates were categorized as shown in Table 1. An unhealthy diet variable dichotomized as candy, cake, chocolate, cocoa and chips intake (<5 days a week; ≥5 days a week) was generated and used in a supplementary analysis.

### 2.5. Statistical Analysis

Statistical analyses were conducted in STATA-16 (StataCorp, College Station, TX, USA). The medians, 5th and 95th pseudo percentiles (average of five observations nearest to the actual percentile) of each semen quality characteristic (volume, sperm concentration, total sperm count, total motility, and morphology) were calculated according to frequency of consumption of sugar-sweetened or artificially sweetened beverages.

We estimated crude and adjusted ratios of semen characteristics according to consumption using negative binomial regression models. This approach was chosen because the conditional distributions were over-dispersed (conditional variance exceeded the conditional mean). The negative binomial regression models were checked by comparing the observed distribution of semen parameters against the model-based distributions from the fitted model using QQ-plots; subsequently, standardized deviance residuals were plotted against model-based predictions. The model check was acceptable (data not shown). The estimates were interpreted as percentage differences between exposure groups. Statistical models were adjusted for the identified potential confounders and the two variables, abstinence time and fever, to increase precision of the estimates. Total motility was additionally adjusted for time from ejaculation to analysis. In the analyses of total sperm count and volume, participants reporting spillage were excluded from the analysis [*n* = 185].

Selection weights were used in all analyses to account for non-participation [49] and derived by multivariable logistic regression of participation (yes; no) on potential predictors of participation (parental socio-economic status, maternal smoking and maternal BMI) to estimate the probability of participation for each participant. These predictors were chosen a priori based on a DAG. The inverse probabilities were used as selection weights. As information from the FEPOS questionnaire was only available for participants; we considered maternal information as proxies for information on the son’s likelihood of participation.

Supplementary analyses were performed with a finer categorization of exposure data (less than weekly; 1–2 days a week; 3–4 days a week; 5–6 days a week; every day) and, in addition, we introduced this new variable as a continuous variable into the regression model. This was undertaken for both sugar-sweetened and artificially sweetened beverage consumption to capture any potential doses-response associations. Additionally, a supplementary analysis for moderate consumption of sugar-sweetened and artificially sweetened beverages combined was performed. The potential mediatory role of BMI was explored by simple adjustment. A supplementary analysis of associations between consumption of sugar-sweetened beverages and semen quality was performed with additional adjustment for an unhealthy diet variable.

### 2.6. Ethical Approval

Informed consent was collected from all participants prior to study participation. The establishment of The FEPOS Cohort was approved by the Scientific Research Ethics Committee for Copenhagen and Frederiksberg (No. H-16015857).

## 3. Results

### 3.1. Study Participants

The participants who consumed sugar-sweetened beverages infrequently (<3 days a week) had candy intake < 3 days a week (63%), cake intake < 3 days a week (66%), consumed energy drinks < 3 days a week (70%), and were normal weight (54%). Similar characteristics were found for participants who had infrequent (<3 days a week) consumption of artificially sweetened beverages. Mothers of participants with infrequent (<3 days a week) consumption of sugar-sweetened beverages were mainly non-smokers (58%) and normal weight (51%). This was also the case for mothers of participants with infrequent (<3 days a week) consumption of artificially sweetened beverages as well as non-smoking mothers of sons with infrequent (<3 days a week) consumption of artificially sweetened beverages (69%) (Table 1).

### 3.2. Beverage Consumption and Semen Quality

In total, 28% of participants consumed sugar-sweetened beverages ≥ 3 days a week and 11% of participants consumed artificially sweetened beverages ≥ 3 days a week. A small group of participants had moderate consumption of both sugar-sweetened and artificially sweetened beverages (*n* = 30). Distributions of semen characteristics according to infrequent or moderate consumption of sugar-sweetened or artificially sweetened beverages are shown in Table 2.

### 3.3. Associations between Exposure and Outcome

Adjusted ratios for semen volume, sperm concentration, total sperm count and total motility between moderate and infrequent consumption of sugar-sweetened or artificially sweetened beverages were all close to one, after adjustment (Table 3). Adjusted ratios for sperm morphology were 11% lower for moderate consumption of artificially sweetened beverages relative to infrequent consumption, with an adjusted ratio of 0.89 (95% CI 0.76, 1.04).

A supplementary analysis with finer categorization of exposure status and with exposure statuses modeled as continuous variables gave results not substantially different from those obtained in the main analysis (Table 4). Neither did a supplementary analysis with both moderate consumption of sugar-sweetened and artificially sweetened beverages combined (data not shown). In a supplementary analysis, further adjustment for BMI did not give substantially different results (Table S1). Supplementary analyses with inclusion of an unhealthy diet variable (Table S2) or presence of varicocele (no; yes; yes, but treated) (Table S3) in the analyses of sugar-sweetened beverage consumption and semen quality parameters did not change the results.

## 4. Discussion

### 4.1. Main Findings

Overall, neither consumption of sugar-sweetened nor artificially sweetened beverages was strongly associated with semen volume, sperm concentration, total motility and total sperm count in this study. The percentage of morphologically normal sperm tended to be lower for moderate than for infrequent consumption of artificially sweetened beverages. However, we did not see any clear dose-response association, and participants with everyday consumption of sugar-sweetened (*n* = 38) or artificially sweetened (*n* = 15) beverages constituted a minority in this study.

### 4.2. Strength and Limitations

The noteworthy strengths of the present study include detailed information on exposures and co-variates collected based on an extensive, online questionnaire that was completed in close proximity to the clinical examination. This allowed for adjustment for potential confounding factors. Further, the study was based on a large population-based sample of young men from the Danish National Birth Cohort [41], which also allowed us to adjust for potential prenatal covariates. Inclusion criteria were limited to ensure a relatively unselected sample of the DNBC participants. However, participants were encouraged to decline participation in case of cryptorchidism, a factor strongly related to semen quality [50].

The cross-sectional study design introduces some limitations. Information on exposure and outcome was collected at one point in time, which prevents the separation of exposure and outcome in time. Participants were instructed to recall consumption of sugar-sweetened and artificially sweetened beverages back in time, which may be imprecise [51,52], potentially leading to measurement error on the exposure. However, this was unlikely to be related to the semen characteristics, as most participants would be unaware of their semen quality due to their young age at recruitment and, hence, low likelihood of participants having tried to reproduce. This reduces the risk of differential measurement error on the exposures and the risk of reverse causation due to the cross-sectional study design. All semen analyses followed WHO guidelines and were performed manually with the technicians blinded to the participants’ exposure, which minimized the risk of measurement error of the outcomes [1]. Internal and external quality control ensured high precision of semen analyses. Biological variation of semen measurement may have caused random measurement error. No physical exam to identify urological diseases was performed, which can be considered a limitation in this study. However, inclusion of varicocele in a supplementary analysis did not impact the findings.

While exposure might be associated with participation, the outcome was likely not due to the young age of participants, limiting the risk of selection bias. However, we cannot rule out that some men opted for participation due to a true suspicion of poor semen quality. Some may be aware of parental fecundability, which might affect the likelihood of participation and potentially introduce selection bias. However, we employed selection weights in all analyses to minimize this potential residual selection problem due to non-participation.

The detailed information on co-variates from the FEPOS questionnaire and the maternal interview during pregnancy in the DNBC [41], allowed us to adjust for potential confounders and strong predictors of the outcome to reduce confounding and improve precision. Although we had solid information on co-variates from both the prenatal and the current period, we did not measure actual, detailed consumption of sugar-sweetened and artificially sweetened beverages from a validated Food Frequency Questionnaire (FFQ).

### 4.3. Interpretation of Results Compared to Existing Literature

The associations have been studied in previous studies. The young age of participants in our study may be part of the explanation of why our results are inconclusive compared to other similar studies [12]. Additionally, consumption of both sugar-sweetened and artificially sweetened beverages was low compared to the studies that found associations between consumption and semen quality [11,13,39]. A cross-sectional study reported a negative association between sugar-sweetened beverage consumption and total motility among 189 healthy, young men from Rochester University [11]. The major strengths of that study included the use of a validated Food Frequency Questionnaire (FFQ) to collect exposure information as basis for derivation of an overall diet variable. However, the study population was small. Men in the highest quartile of sugar-sweetened beverage consumption had a daily intake of ≥ 1.3 servings. Hence, the exposure contrast was possibly much higher than in our study or might indicate risk of exposure misclassification in our study. This study found no evidence regarding BMI as an intermediate factor of the association. In addition, a recently published study by Nassan et al. found intake of sugar-sweetened beverages to be associated with lower sperm concentration and lower total sperm count [13]. The participants were the same age as in our study, but consumption of sugar-sweetened beverages was much higher compared to our study.

A cross-sectional study also by Nassan et al. investigated associations between dietary patterns and testicular function in 2935 young Danish men (median age 19 years) [53]. They found that higher adherence to a Western diet pattern (high intake of sugar-sweetened beverages, sweets, desserts, french fries, pizza, processed food, energy drink etc.) was associated with lower semen quality. Nassan et al. used a modified version of a previously validated Food Frequency Questionnaire (FFQ). Hence, they were able to determine that the highest quintile of young men, who mainly had a Western diet pattern, had a total sugar intake of 90 g a day. Similar information was not available in our study. The majority of the participants in our study did not have a Western diet pattern. In general, their diet patterns more resembled with what Nassan et al. [53] called the prudent pattern and the open-sandwich pattern (healthier diet patterns). The diet pattern together with the low consumption of sugar-sweetened and artificially sweetened beverages among our study population might explain the differences between the two studies.

A cohort study with prospectively collected exposure data also investigated consumption of sugar-sweetened beverages [12]. However, compared to our study, the outcome measure was fecundability. The target population was pregnancy planners, which were probably aware of their fecundability, hence, introducing risk of selection bias. The study population consisted of both females and their male partners, who were followed from 2013–2017. Consumption of sugar-sweetened beverages 7 days a week by, in the male partner were associated with lower fecundability. As in the cross-sectional study described above, the exposure contrast was much larger compared to our study. In our study, only a minority reported consumption of sugar-sweetened beverages every day, as the majority of participants in our study consumed sugar-sweetened beverages < 3 days a week (72%) and artificially sweetened beverages < 3 days a week (89%). Based on data reported in the FEPOS cohort, we can therefore not exclude that higher consumption might affect semen quality negatively. Hatch et al. found no evidence of association between consumption of artificially sweetened beverages and fecundability. In our study, there were indications of reduced morphology in moderate consumers of artificially sweetened beverages. Morphology, in particular, was not a priori hypothesized to play a significant role. We cannot exclude that the changes found for morphology might be a chance finding. Gong et al. and Anbara et al. found evidence of associations between intake of saccharin and aspartame on testis function in mice, but no particular effect on morphology was found [38,39,40].

Caffeine (especially from cola) was associated with reduced sperm concentration and total sperm count in a Danish study investigating caffeine intake and semen quality in a study population of 2554 young men [14]. In the present study, we did not have an overall caffeine variable. However, our analyses were adjusted for energy drink and coffee consumption. Cola was part of the sugar-sweetened beverage consumption among participants, but we were not able to determine the precise amount.

## 5. Conclusions

In conclusion, we did not observe marked associations between consumption of sugar-sweetened or artificially sweetened beverages and semen quality in young men, although a slightly lower percentage of morphologically normal sperm was observed among consumers of artificially sweetened beverages ≥ 3 days per week in this study. The study population had only moderate consumption of sugar-sweetened and artificially sweetened beverages compared to similar studies. It is reassuring for young people that moderate consumption of sugar-sweetened or artificially sweetened beverages appears to have limited effects on semen quality, but the finding should be interpreted cautiously and needs confirmation in future studies. Further large-scale studies with higher exposure levels and more precise estimation of consumption of sugar-sweetened and artificially sweetened beverages are warranted.

## Figures and Tables

**Table 1:** Characteristics of the 1047 participants sorted by beverage consumption, with available information on sugar-sweetened, artificially sweetened beverage consumption and semen parameters, and maternal characteristics, FEPOS 2017–2019.

Participants’ Characteristics (2017–2019)	Sugar-Sweetened Beverage Consumption ^a^		Artificially Sweetened Beverage Consumption ^a^	
	<3 Days a Week	≥3 Days a Week	<3 Days a Week	≥3 Days a Week
Candy intake				
<3 days a week	658 (63)	199 (19)	769 (73)	88 (8)
≥3 days a week	99 (9)	91 (9)	165 (16)	25 (3)
Cake intake				
<3 days a week	689 (66)	246 (24)	841(80)	94 (9)
≥3 days a week	68 (6)	44 (4)	93 (9)	19 (2)
Cocoa intake				
Less than weekly	587 (56)	203 (19)	709 (68)	79 (8)
Weekly	172 (17)	87 (8)	225 (21)	34 (3)
Energy drink intake				
<3 days a week	734 (70)	234 (22)	876 (84)	92 (9)
≥3 days a week	23 (3)	56 (5)	58 (5)	21 (2)
Coffee intake				
<3 days a week	303 (35)	142 (17)	394 (46)	51 (6)
≥3 days a week	323 (37)	94 (11)	373 (43)	44 (5)
Smoking status				
Yes	267 (26)	146 (14)	357 (34)	56 (5)
No	144 (14)	490 (46)	577 (56)	57 (5)
Body Mass Index				
Underweight	53 (5)	25 (3)	72 (7)	6 (1)
Normal weight	571 (54)	203 (19)	701 (67)	73 (7)
Overweight	133 (13)	62 (6)	161 (15)	34 (3)
Spillage				
Yes	132 (12)	50 (5)	160 (15)	22 (2)
No	620 (60)	239 (23)	770 (74)	89 (9)
Abstinence time				
<2 h	256 (25)	107 (10)	326 (31)	37 (4)
2–4 h	388 (37)	158 (15)	482 (46)	64 (6)
>4 h	113 (11)	25 (2)	126 (12)	12 (1)
Fever				
Yes	102 (10)	36 (3)	121 (12)	17 (2)
No	570 (54)	215 (21)	705 (67)	80 (7)
Don’t know	85 (8)	39 (4)	108 (10)	16 (2)
Time to analysis				
<60 min	562 (54)	216 (21)	690 (66)	88 (8)
≥60 min	188 (18)	72 (7)	236 (24)	24 (2)
Maternal Characteristics (1997–2000)				
Maternal smoking status				
Yes	155 (15)	88 (8)	214 (20)	29 (3)
No	602 (58)	202 (19)	720 (69)	84 (8)
Maternal Body Mass Index				
Underweight	69 (7)	28 (3)	92 (8)	6 (1)
Normal weight	525 (51)	191 (19)	647 (63)	69 (7)
Overweight	146 (14)	63 (6)	172 (17)	37 (4)
Socio Economic Status (highest of parents)				
Student or economically inactive	42 (4)	7 (1)	43 (4)	0 (0)
Skilled or unskilled worker	193 (18)	106 (10)	263 (25)	36 (3)
Low grade professional	242 (23)	102 (10)	303 (29)	41 (4)
High grade professional	280 (27)	75 (7)	320 (32)	35 (3)

Displayed as numbers of participants, *n* (%); ^a^ Data on participants appear twice, once for each exposure group.

**Table 2:** Distribution of semen parameters according to low or high consumption of sugar-sweetened and artificially sweetened beverages, FEPOS 2017–2019.

Semen Parameters (Medians, 5th, 95th Percentiles) ^b^	Sugar-Sweetened Beverage Consumption ^a^			Artificially Sweetened Beverage Consumption ^a^		
	N	Infrequent (<3 Days a Week)	Moderate (≥3 Days a Week)	N	Infrequent (<3 Days a Week)	Moderate (≥3 Days a Week)
Semen volume (mL)	1047	2.7 (1.9, 3.7)	2.6 (1.8, 3.6)	1047	2.7 (1.9, 3.7)	2.7 (1.8, 3.6)
Sperm concentration (mill./mL)	1047	39.7 (18.7, 73.2)	33.7 (18.8, 68.0)	1047	38.9 (19.0, 72.8)	34.6 (16.6, 65.3)
Total motility (A + B%)	1031	73.0 (64.0, 80.0)	71.4 (63.0, 79.0)	1031	72.3 (63.0, 80.0)	72.4 (66.8, 80.3)
Sperm morphology (% normal sperm)	1026	6.0 (0.3, 10.0)	6.0 (2.9, 10.0)	1026	6.0 (3.0, 10.0)	6.0 (3.5, 9.7)
Total sperm count (mill.)	1047	104.2 (47.9, 203.9)	94.3 (39.4, 180.2)	1047	103.3 (45.8, 202.3)	96.9 (39.8, 167.4)

^a^ Data on participants appear twice, once for each exposure group; ^b^ Medians and percentiles shown as pseudo-percentiles (an average of 5).

**Table 3:** Crude and adjusted outcome ratios according to sugar-sweetened and artificially sweetened beverage consumption among participants, FEPOS 2017–2019.

Semen Parameters	Sugar-Sweetened Beverage Consumption Unadjusted	Sugar-Sweetened Beverage Consumption Adjusted		Artificially Sweetened Beverage Consumption Unadjusted	Artificially Sweetened Beverage Consumption Adjusted	
	Crude Ratio ^a^	Adjusted Ratio ^a,b^	95% CI	Crude Ratio ^a^	Adjusted Ratio ^a,b^	95% CI
Semen volume ^c^	0.97	0.99	(0.91, 1.08)	1.02	1.03	(0.92, 1.17)
Sperm concentration	0.92	1.03	(0.90, 1.19)	0.89	0.96	(0.81, 1.15)
Total motility ^d^	0.99	0.98	(0.95, 1.02)	1.01	1.01	(0.98, 1.05)
Sperm morphology (% normal sperm)	0.99	0.99	(0.87, 1.12)	0.90	0.89	(0.76, 1.04)
Total sperm count ^c^	0.86	0.98	(0.84, 1.15)	0.89	0.99	(0.81, 1.21)

^a^ Ratio between the semen quality characteristics for high consumption relative to low consumption; ^b^ Adjusted for candy intake, cake intake, energy drink consumption, coffee consumption, smoking, maternal smoking, maternal BMI, parental socio-economic status, abstinence time and fever; ^c^ Participants with recording of spillage excluded from analyses on semen volume and total sperm count (mill.); ^d^ Analyses on total motility (A + B%) additionally adjusted for time to analysis.

**Table 4:** Adjusted outcome ratios according to sugar-sweetened and artificially sweetened beverage consumption among participants, FEPOS 2017–2019.

Semen Parameters	Sugar-Sweetened Beverage Consumption Adjusted			Artificially Sweetened Beverage Consumption Adjusted		
		Ratio ^a,b^	95% CI		Ratio ^a,b^	95% CI
Semen volume ^c^	Less than weekly (n = 362)	1		Less than weekly (n = 869)	1	
	1–2 days a week (n = 485)	0.97	(0.90, 1.05)	1–2 days a week (n = 171)	1.04	(0.95, 1.14)
	3–4 days a week (n = 208)	0.94	(0.84, 1.05)	3–4 days a week (n = 82)	1.05	(0.91, 1.22)
	5–6 days a week (n = 74)	1.05	(0.91, 1.22)	5–6 days a week (n = 30)	1.05	(0.87, 1.26)
	Every day (n = 38)	0.98	(0.81, 1.18)	Every day (n = 15)	0.82	(0.56, 1.20)
	* Continuous	1.00	(0.98, 1.02)	* Continuous	1.01	(0.98, 1.04)
Sperm concentration	Less than weekly (n = 362)	1		Less than weekly (n = 869)	1	
	1–2 days a week (n = 485)	1.02	(0.89, 1.17)	1–2 days a week (n = 171)	1.02	(0.86, 1.20)
	3–4 days a week (n = 208)	1.05	(0.87, 1.27)	3–4 days a week (n = 82)	0.96	(0.77, 1.20)
	5–6 days a week (n = 74)	1.04	(0.81, 1.33)	5–6 days a week (n = 30)	1.03	(0.78, 1.36)
	Every day (n = 38)	0.99	(0.69, 1.42)	Every day (n = 15)	0.81	(0.47, 1.41)
	* Continuous	1.00	(0.97, 1.04)	* Continuous	0.99	(0.95, 1.03)
Total motility ^d^	Less than weekly (n = 362)	1		Less than weekly (n = 869)	1	
	1–2 days a week (n = 485)	1.00	(0.97, 1.03)	1–2 days a week (n = 171)	1.00	(0.97, 1.04)
	3–4 days a week (n = 208)	0.98	(0.94, 1.02)	3–4 days a week (n = 82)	1.00	(0.96, 1.05)
	5–6 days a week (n = 74)	0.99	(0.94, 1.04)	5–6 days a week (n = 30)	1.03	(0.98, 1.09)
	Every day (n = 38)	0.95	(0.87, 1.03)	Every day (n = 15)	1.02	(0.90, 1.15)
	* Continuous	0.99	(0.99, 1.00)	* Continuous	1.00	(0.99, 1.01)
Sperm morphology (% normal sperm)	Less than weekly (n = 362)	1		Less than weekly (n = 869)	1	
	1–2 days a week (n = 485)	0.92	(0.81, 1.05)	1–2 days a week (n = 171)	0.93	(0.81, 1.06)
	3–4 days a week (n = 208)	0.95	(0.80, 1.13)	3–4 days a week (n = 82)	0.89	(0.74, 1.09)
	5–6 days a week (n = 74)	0.92	(0.72, 1.16)	5–6 days a week (n = 30)	0.86	(0.67, 1.11)
	Every day (n = 38)	0.92	(0.68, 1.25)	Every day (n = 15)	0.86	(0.50, 1.49)
	* Continuous	0.99	(0.96, 1.02)	* Continuous	0.97	(0.93, 1.01)
Total sperm count ^c^	Less than weekly (n = 362)	1		Less than weekly (n = 869)	1	
	1–2 days a week (n = 485)	1.08	(0.93, 1.24)	1–2 days a week (n = 171)	0.97	(0.83, 1.13)
	3–4 days a week (n = 208)	1.00	(0.81, 1.22)	3–4 days a week (n = 82)	0.97	(0.74, 1.26)
	5–6 days a week (n = 74)	1.12	(0.86, 1.45)	5–6 days a week (n = 30)	1.08	(0.82, 1.41)
	Every day (n = 38)	0.99	(0.67, 1.46)	Every day (n = 15)	0.66	(0.36, 1.23)
	* Continuous	1.00	(0.96, 1.04)	* Continuous	0.99	(0.95, 1.04)

^a^ Reference: Less than weekly ^b^ Adjusted for candy intake, cake intake, energy drink consumption, coffee consumption, smoking, maternal smoking, maternal BMI, parental socio-economic status, abstinence time and fever ^c^ Participants with recording of spillage excluded from analyses on semen volume and total sperm count (mill.) ^d^ Analyses on total motility (A + B%) additionally adjusted for time to analysis * Continuous: Relative change in semen characteristics per 1 category change in weekly consumption.

## Supplementary Materials

The following are available online at https://www.mdpi.com/article/10.3390/ijerph19020682/s1, Table S1: Adjusted outcome ratios with and without BMI adjustment according to sugar-sweetened and artificially sweetened beverage consumption among participants, FEPOS 2017–2019, Table S2: Adjusted outcome ratios including unhealthy diet according to sugar-sweetened beverage consumption among participants, FEPOS 2017–2019; Table S3: Adjusted outcome ratios including varicocele according to sugar-sweetened or artificially sweetened beverage consumption among participants, FEPOS 2017–2019.
