# Supplementary material for: Consumption of Sugar-Sweetened or Artificially Sweetened Beverages and Semen Quality in Young Men: A Cross-Sectional Study
Source: Int J Environ Res Public Health. 2022 Jan 7;19(2):682. doi: 10.3390/ijerph19020682 (PMC8775653; doi:10.3390/ijerph19020682)
Supplement: Supplementary file 1 [file ijerph-19-00682-s001.zip › ijerph-1513320-supplementary.pdf]

Table S1: Adjusted outcome ratios with and without BMI adjustment according to sugar-sweetened and artificially sweetened beverage consumption among participants, FEPOS 2017-2019

Semen parameters	**Sugar-sweetened beverage consumption** Adjusted ratio and 95% CI[a,b]	**Sugar-sweetened beverage consumption** Adjusted ratio and 95% CI[a,c]	**Artificially sweetened beverage consumption** Adjusted ratio and 95% CI[a,b]	**Artificially sweetened beverage consumption** Adjusted ratio and 95% CI[a,c]
Semen volume[d]	0.98 (0.91, 1.07)	0.98 (0.90, 1.07)	1.04 (0.93, 1.17)	1.04 (0.93, 1.18)
Sperm concentration	1.02 (0.89, 1.17)	1.02 (0.89, 1.17)	0.97 (0.81, 1.16)	0.97 (0.81, 1.15)
Total motility[e]	0.98 (0.95, 1.02)	0.98 (0.95, 1.02)	1.01 (0.98, 1.05)	1.01 (0.98, 1.05)
Sperm morphology (% normal sperm)	1.02 (0.91, 1.15)	0.99 (0.88, 1.13)	0.89 (0.76, 1.04)	0.88 (0.76, 1.03)
Total sperm count[d]	0.98 (0.84, 1.14)	0.98 (0.84, 1.14)	0.99 (0.81, 1.21)	1.00 (0.82, 1.23)

[a]Ratio between the semen quality characteristics for moderate consumption (≥3 days per week) relative to infrequent consumption (<3 days per week)

[b]Adjusted for candy intake, cake intake, energy drink consumption, coffee consumption, smoking, maternal smoking, maternal BMI, parental socio-economic status, abstinence time and fever

[c] Adjusted for BMI, candy intake, cake intake, energy drink consumption, coffee consumption, smoking, maternal smoking, maternal BMI, parental socio-economic status, abstinence time and fever

[d]Participants with recording of spillage excluded from analyses on semen volume and total sperm count (mill.)

[e]Analyses on total motility (A + B %) additionally adjusted for time to analysis

Table S2: Adjusted outcome ratios including unhealthy diet according to sugar-sweetened beverage consumption among participants, FEPOS 2017-2019

Semen parameters	**Sugar-sweetened beverage consumption** Adjusted ratio and 95% CI[a,b]
Semen volume[c]	1.00 (0.93, 1.08)
Sperm concentration	1.00 (0.88, 1.13)
Total motility[d]	0.98 (0.96, 1.01)
Sperm morphology (% normal sperm)	1.00 (0.90, 1.11)
Total sperm count[c]	0.96 (0.84, 1.01)

[a]Ratio between the semen quality characteristics for moderate consumption (≥3 days per week) relative to infrequent consumption (<3 days per week)
[b]Adjusted for unhealthy diet, energy drink consumption, coffee consumption, smoking, maternal smoking, maternal BMI, parental socio-economic status, abstinence time and fever
[c]Participants with recording of spillage excluded from analyses on semen volume and total sperm count (mill.)
[d]Analyses on total motility (A + B %) additionally adjusted for time to analysis

Supplementary Table S3: Adjusted outcome ratios including varicocele according to sugar-sweetened or artificially sweetened beverage consumption among participants, FEPOS 2017-2019

Semen parameters	**Sugar-sweetened beverage consumption** Adjusted ratio and 95% CI[a,b]	**Artificially sweetened beverage consumption** Adjusted ratio and 95% CI[a,b]
Semen volume[c]	0.99 (0.91, 1.07)	1.03 (0.92, 1.16)
Sperm concentration	1.03 (0.89, 1.18)	0.96 (0.81,1.14)
Total motility[d]	0.98 (0.95, 1.02)	1.01 (0.98, 1.05)
Sperm morphology (% normal sperm)	0.98 (0.86, 1.11)	0.89 (0.77, 1.04)
Total sperm count[c]	0.97 (0.83, 1.14)	0.98 (0.80, 1.20)

[a]Ratio between the semen quality characteristics for moderate consumption (≥3 days per week) relative to infrequent consumption (<3 days per week)
[b]Adjusted for candy intake, cake intake, energy drink consumption, coffee consumption, smoking, maternal smoking, maternal BMI, parental socio-economic status, abstinence time, varicocele, and fever
[c]Participants with recording of spillage excluded from analyses on semen volume and total sperm count (mill.)
[d]Analyses on total motility (A + B %) additionally adjusted for time to analysis
